# Health on the Banks of the Rio Grande

**DOI:** 10.1289/ehp.113-a304

**Published:** 2005-05

**Authors:** Julia R. Barrett

At the New Mexico Center for Environmental Health Sciences (NMCEHS), community concerns and relationships are as much at the center’s foundation as the latest research techniques. According to center director Scott Burchiel, the unique populations of New Mexico—including a high number of Hispanics and Native American tribes, pueblos, and sovereign nations—steer the center’s course. “One of the things we have tried to do is to determine what these populations’ concerns are for environmental health, because we want our center to be focused on environmental public health, and we want to work on basic research projects that address issues that people in this region are particularly interested in,” he says.

Based in Albuquerque as a partnership of The University of New Mexico Health Sciences Center and the Lovelace Respiratory Research Institute (LRRI), the NIEHS-funded center comprises major research cores in environmental cancer and oxidative stress, cardiovascular toxicology, environmental lung disease, and population health and epidemiology. The center also includes world-class facility cores (particularly for inhalation toxicology), a pilot project program to provide funding for small innovative research studies, and an unusually strong community outreach and education program (COEP).

Community input is essential, as is trust. “We have investigators who may have certain interests for certain kinds of projects and want to work with communities,” says Burchiel. “But when we go to the community, we find out that [the community has] a different agenda, different interests. We’ve been learning how to work with communities since we’ve had this environmental health center.” It’s a process that involves a lot of development of community relationships and trust relationships.

Burchiel says that investigating environmental health problems such as asthma requires a broad definition of *environment*. He says, “In our center, we define the environment quite broadly. It includes nutritional, social, behavioral, economic, and built environment components. . . . We have not only environmental epidemiologists [but] also social epidemiologists who help us look at social factors in assessing economic and poverty factors.”

## The Heart (and Lungs) of the Matter

The center originated in 1998 with a three-year planning grant focused on environmental lung disease and Native Americans. Of particular interest was asthma incidence among Native American schoolchildren. At one pueblo, for example, 11.3% of children between ages 3 and 13 had been diagnosed with asthma, an incidence that was more than twice the national average for the same age group. Follow-up research focused on potential causes, including exposures to endotoxin and wood smoke, but Burchiel indicates that there are still no definitive conclusions.

Center investigators are also investigating asthma in an urban setting as well. The NMCEHS is currently pursuing an NIEHS Advanced Research Cooperation in Environmental Health grant with the University of Texas at El Paso to investigate factors that may affect asthma incidence among El Paso schoolchildren. Approximately 700,000 people reside in El Paso, and the neighboring Mexican city of Cuidad Juárez boosts the urban population to about 2 million. Old cars, unregulated trash burning, brick kilns, smelters, and heavy, slow-moving diesel-truck traffic crossing at the border and *maquiladora* industries along the U.S.–Mexican border contribute to significant air quality problems. Winter-time atmospheric inversions, which prevent air pollutants from circulating away from the cities, compound the problems. The grant project will draw on the LRRI’s expertise in environmental assessment and the university Health Sciences Center’s clinical expertise.

The inhalation toxicology facilities of the LRRI also support center investigations in environmental cardiology, a relatively new discipline. “One of the things that’s fairly interesting that’s come out in the last five to ten years is that environmental pollutants may be contributing to both the incidence as well as the severity and progression of cardiovascular disease in the United States,” says Mary Walker, director of the cardiovascular toxicology core. She notes that epidemiological air pollution studies, despite focusing on pulmonary disease and asthma, have yielded intriguing information about potential effects on the heart of particulate matter, ozone, carbon monoxide, and other pollutants.

Walker studies how such pollutants might affect cardiac birth defects as well as how fetal exposures might relate to cardiovascular disease in adulthood. A current project follows up on findings from an epidemiological study published in the January 2002 *American Journal of Epidemiology*. In that study, researchers from the University of California, Los Angeles, found fairly strong correlations between maternal air pollution exposure during the first trimester of pregnancy and children being born with overt heart defects. “This was something that I don’t think anyone would have predicted,” says Walker.

She and her colleagues are now collaborating with LRRI researchers to develop an animal model to test the observations that came out of that human epidemiology study. Their model uses diesel exhaust as the air pollutant of interest and also includes the biggest contributors to air pollution such as particulate matter, polycyclic aromatic hydrocarbons, and carbon monoxide.

“Our current hypothesis is that it’s actually the carbon monoxide component of the diesel exhaust that leads to a low-oxygen, or hypoxic, condition in the developing fetal heart,” says Walker, citing recent research at Case Western Reserve University. In that study, published in the March 2004 issue of *Developmental Biology*, researchers showed that normal septation, or formation of the aorta from the pulmonary artery, relies on hypoxia as a necessary transcriptional signal during heart development. “What we hypothesize is that the carbon monoxide component of the diesel exhaust is actually making that environment even more hypoxic and disrupting normal gene programming that should lead to normal septation of the aorta and pulmonary artery,” says Walker.

Walker’s team will look at factors such as expression of genes regulated by hypoxia in the developing heart and whether expression is altered in response to diesel exhaust exposure. She is also interested in subtle changes in the heart or in gene programming that may occur during fetal development and underlie disease in adulthood.

## Kidney Toxicants in the Home

Environmental factors that may enhance disease progression is also a theme in research conducted on the high incidence of nondiabetic kidney disease among Native Americans in New Mexico. A genetic link is suspected; prior to the center’s formation, University of New Mexico researchers were already working with residents of a rural Native American community to identify the potential root cause or causes of the high incidence. Residents at this pueblo have a very high prevalence of kidney disease and a very high prevalence of diabetes, says Burchiel. Compared with other Native Americans, these pueblo residents experience kidney disease at more than a 5-fold higher incidence; compared with European Americans, incidence is more than 18-fold higher.

Although Native American tribes generally do not permit genetic research on their members for legal and cultural reasons, this pueblo is an exception. “The [participants] are very unusual in that they’ve been working with The University of New Mexico and a group of national investigators,” says Burchiel. The key reason for this unique participation is the high incidence of kidney disease—virtually every pueblo resident has a family member or knows someone who has died or is dying from this disease. The residents understand that there is likely a genetic component. Therefore, they have decided to take the unusual step of participating in genetic research.

With the formation of the NMCEHS, new avenues of research opened as investigators began considering gene–environment interactions in kidney disease. In considering potential environmental triggers, investigators began to focus on jewelry making—a common cottage industry in the Native American population—and whether people were being exposed to metals through their in-home jewelry production.

“Many of the metals found in jewelry making products are potentially toxic to the kidney,” says Melissa Gonzales, a member of the center’s population health and epidemiology core. This poses serious risks for a population that may already be genetically predisposed to develop kidney disease. In a preliminary study, Gonzales analyzed house dust samples for metal contaminants, including cadmium, nickel, copper, tin, lead, and silver. She found a relatively high concentration of metals in the dust in homes where jewelry was made compared to homes where no jewelry was made. The study also involved a survey and inventory of products used in jewelry making, which helped correlate the presence of metals with the work done in the home.

Gonzales explains that the main metal used in the residents’ jewelry making is sterling silver—an alloy of silver, copper, and nickel—but the inventory of related materials provided an explanation for unexpected findings such as boron, which is used in the form of powdered boric acid as a protective coating during the jewelry-making process. Researchers also provided study participants with information extracted from material safety data sheets on how to use the materials safely. The researchers are currently applying for a research grant based in part on the results of this study.

## Naturally Occurring Threats

Naturally occurring substances fuel other populationwide environmental health concerns in New Mexico. In the U.S. Southwest, drinking water drawn from underground sources may have high concentrations of arsenic due to leaching of the mineral from surrounding rock over the course of millions of years. New Mexico has some of the highest drinking water concentrations of arsenic in the United States. Arsenic can induce cancers of the bladder, lung, skin, and other organs.

“Over the last five to ten years, there’s been an increased interest in arsenic research, partly because people realized arsenic is an important carcinogen, partly because of the political debate,” says Ke Jian Jim Liu, coleader of the environmental cancer and oxidative stress core. The political debate to which he refers centers on the U.S. Environmental Protection Agency’s new drinking water standard for arsenic of 10 parts per billion (ppb) or less by 2006. Most U.S. water supplies met the old standard of 50 ppb, but the new standard places some out of compliance. For example, according to the City of Albuquerque’s 2003 water quality report, some areas of the city had water containing up to 24 ppb of arsenic.

Arsenic is not a very strong carcinogen. However, exposure via drinking water is chronic and unavoidable, and Liu describes an additional risk factor due to the state’s high altitude and wealth of sunshine: “There have been reports that ultraviolet radiation and arsenic synergistically increase the rate of skin cancer development, so arsenic has become an even more important issue for the people in the Southwest.” A review article on this topic appeared in the August 2004 issue of *Toxicology and Applied Pharmacology*. No one is certain what the mechanism is for this synergistic action, although Liu hypothesizes that arsenic may inhibit repair of ultraviolet-induced DNA damage.

Several arsenic-related projects are under way at the center, and each aspect of arsenic research—neuroscience, toxicology, carcinogenesis, epidemiology—is represented by a different group that brings its own unique set of strengths to the topic. One group is conducting an epidemiological assessment of arsenic exposure and cancer rates among Southwesterners. Another group is focusing on potential neurological effects and has preliminary animal data that suggest a link between arsenic exposure and decreased cognitive abilities in infancy and early childhood. Liu’s own research focuses on identifying the arsenic-induced events that lead a normal cell to become cancerous. “Our hope is that by focusing on several areas this may lead to some interesting findings and potentially provide some insight on how arsenic induces skin cancer and some guidance on development of therapeutic interventions,” he says.

Another naturally occurring threat arises from New Mexico’s rich mineral resources. Uranium represents both naturally occurring and occupational exposures—as well as a keystone environmental justice issue. New Mexico boasts the largest open-pit uranium mine in the world, the Jackpile mine, along with many smaller mines. Much of the mining workforce has been drawn from Native American communities, and there is concern for both the workers’ health as well as potential widespread environmental contamination from abandoned mines.

## A Partnership in Trust

Uranium exposure is one of the key issues addressed by community outreach and education efforts at the NMCEHS. COEP staff work closely with 20 chapters (local tribal government units) of the Navajo Nation to provide training to tribal members on environmental health, survey methodology, and water sampling. The goal of the training is to build the chapters’ capacity to understand and make informed decisions specifically about the effects of uranium exposure on kidney health. As this partnership has developed, the COEP has involved other center members—including biostatisticians, modelers, and epidemiologists—to support community-driven research on the relationship between kidney disease and uranium exposures.

The COEP is also beginning a similar effort with the Cheyenne River Sioux Tribe of South Dakota, at that tribe’s request. This effort will focus on building environmental health capacity to evaluate the impacts of mercury contamination of surface waters on tribal lands, as well as other environmental justice concerns.

The COEP also reaches out to local youth to show how they can be potent advocates for the environment. In February 2004, COEP staff worked with the South Valley Partners for Environmental Justice (a nonprofit partnership between Bernalillo County, the Rio Grande Community Development Corporation, and the COEP) and Amigos Bravos—Friends of the Wild Rivers to provide a three-part training course to staff at the local Indio-Hispano Academy of Agricultural Arts and Sciences. The academy provides a program in which at-risk youth (many of whom are referred by the courts as an alternative to juvenile detention) become involved in activities that focus on protecting and preserving the cultural and traditional agricultural lifestyles of the surrounding region. The partnership training taught the staff about the provisions of the Clean Water Act, surface and groundwater quality and hydrology, contaminant transport, basic toxicology, and potential surface water exposure pathways.

After hearing about the training from their mentors, seven academy students felt that the portion of the Rio Grande that passes through Albuquerque had been wrongly classified by the Environmental Protection Agency as “secondary contact,” meaning residents generally do not have direct bodily contact with the water. The students provided critical testimony to policy makers in Santa Fe during the New Mexico Water Quality Control Commission’s Triennial Review that spring, describing historical and current community uses of the *acequias* (irrigation waters from the Rio Grande) for farming, fishing, and swimming. They provided photo documentation of these uses.

The testimony resulted in the October 2004 decision by policy makers to raise the classification of that portion of the river to primary rather than secondary contact, meaning the water must be clean enough to permit activities involving direct contact with the water. This classification will result in more stringent standards and lower permissible levels of fecal coliform in the water.

## A Community Resource

COEP director Johnnye Lewis describes the outreach program’s philosophy as building relationships and trust for the long term. “If we are doing our jobs,” she says, “communities can rely on us as a resource to help them answer their own questions by making environmental health research and decision making understandable and accessible.”

In truth, that philosophy extends to the entire center. “We’re really trying to work on—as we say in our mission statement—our regionally relevant environmental public health issues,” says Burchiel. As a measure of the center’s success and the trust it has built, surrounding communities have come to rely on center staff for answers to environmental health concerns. “Now, whenever there’s a question about environmental health, we get called,” says Burchiel. “That’s very gratifying.”

## Figures and Tables

**Figure:**
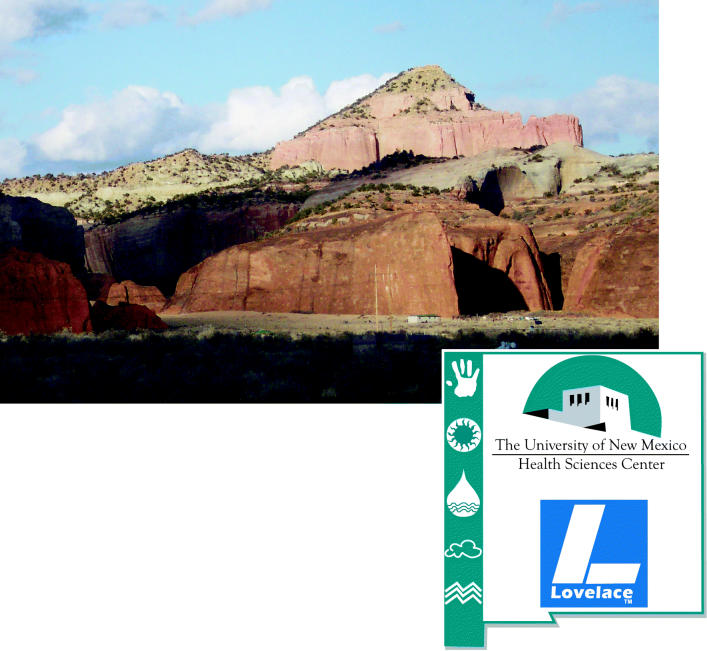
**The lay of the land.** A number of Native American pueblos make up part of the unique constituency served by the New Mexico Center for Environmental Health Sciences.

**Figure:**
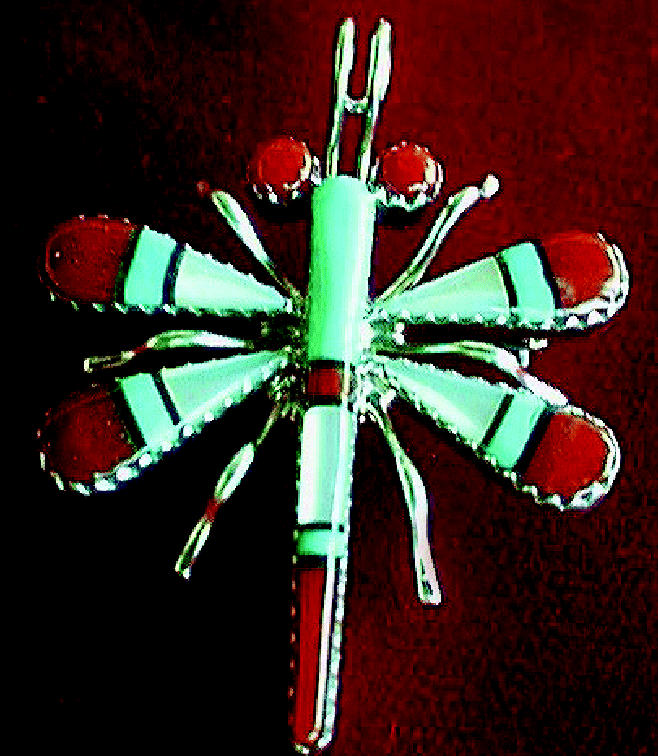
**Risky business.** In-home jewelry making, a common cottage industry among Native Americans, exposes community members to metals that may trigger a genetic predisposition to kidney disease.

**Figure:**
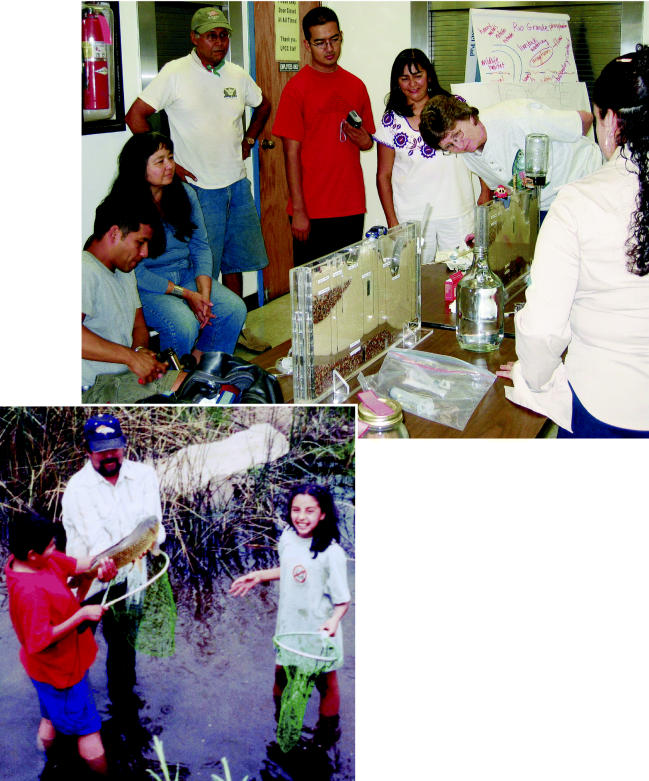
**Knowledge is power.** Staff at the Indio-Hispano Academy of Agricultural Arts and Sciences (above) received training on the Clean Water Act, surface and groundwater quality and hydrology, contaminant transport, basic toxicology, and potential surface water exposure pathways. This new knowledge empowered academy youth to challenge the “secondary contact” designation assigned to the portion of the Rio Grande running through Albuquerque. The students convinced policy makers that this portion of the river actually requires the more stringent “primary contact” designation due to human uses of the river (left).

